# Becoming fully circular facilitated by PAT

**DOI:** 10.1007/s00216-025-05977-y

**Published:** 2025-06-27

**Authors:** Tobias Eifert, Martin Gerlach, Katharina Dahlmann, Bernhard Lendl, Matthias Rädle, Martin Jaeger

**Affiliations:** 1 Covestro, Uerdingen, Krefeld, Germany; 2 AK PAT, GDCh, Frankfurt Am Main, Germany; 3 Bayer, Dormagen, Germany; 4 Domat/Ems, Hamilton, Switzerland; 5 TU Wien, Vienna, Austria; 6 HS Mannheim, Mannheim, Germany; 7 HS Niederrhein, Krefeld, Germany

**Keywords:** Process analytical technology, Solid-phase extraction ion-chromatography conductivity, Quantum cascade laser vibrational circular dichroism, Single-use technology, Infrared spectroscopy

## Abstract

Process analytical technologies (PAT) have contributed to helping industrial processes become more efficient, more sustainable, safer, and more reliable for more than two decades. Real-time monitoring is the key to process control but also to process knowledge. Advancements in instrument performance and in the availability of complex data analysis methodologies have made PAT a fundamental pillar in quality by design concepts. On account of the 20th anniversary of the Arbeitskreis PAT of the GDCh, this article assembles use cases for and from PAT applications that do or will contribute to and impact relevant and forward-looking processes. Five examples are presented for the monitoring of sustainable processes from the recycling of industrial process water, solvents, and plastics, from biotechnological production using disposable reactors, i.e., single-use technology, and of product safety confirmation amenable to pharmaceutical production and real-time release. The applied PAT methodology illustrates use cases of online solid-phase extraction, ion-chromatography conductivity determination, mid- and near-infrared spectroscopy, and external cavity quantum cascade laser vibrational circular dichroism. The reviewed examples are taken to a perspective on future PAT developments.

**Graphical Abstract:**

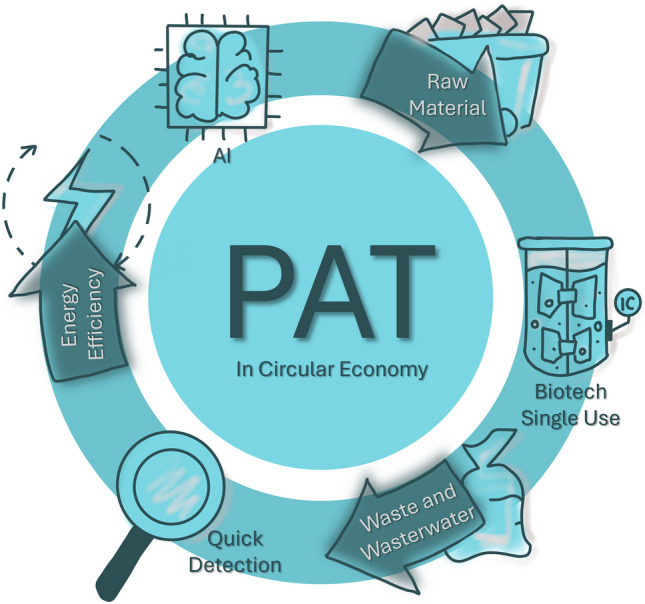

## Introduction

Some fifteen years ago, Chemistry decided to endeavor on its challenging journey to become Green and Sustainable [1]. Simultaneously, analytical chemistry faced an endo and an exo role: to grow green and to support chemistry on its transformation path [2, 3]. From a wider perspective, the promising strategy toward sustainability lies in the transformation of our economy into a circular economy along with resourceful and efficient processes. Alternative raw materials, platform chemicals, innovative recycling, upcycling, carbon footprint, and renewable energies form the heart of the new economical approach. Biotechnological processes are often seen as preferential [4]. Biobased feedstocks and substrate streams from recycling are often associated with a higher composition variability than traditional processes. The resulting modifications of existing processes and the design of new ones will heavily rely on process analytical technologies (PAT) but will also exercise a major impact on their performance, application, and development. Further, PAT needs to monitor the higher variability to allow downstream processes or at least reduce the impact of complex compositions on them.

Green PAT—in both roles—is heading even more rapidly toward fast monitoring methodology, multi-information data streams, artificial intelligence (AI) or machine learning (ML) data analysis, feedback, and feedforward process control [5]. With an expected shift to continuous processes, real-time monitoring will become even more important. Complementarily, single-use batch reactors for biotechnological processes shall require flexible, yet robust PAT solutions [6]. The number and amount of components in circularly conducted processes may grow with time such that a wider range of information is constantly needed to acquire and possess process knowledge sufficient for instantaneous control. Hence, spectroscopic methodology will gain in importance, where the value of contribution will be based on current and long-term technological advances. These may be very different for the various sub-technologies. Current trends have been summarized recently [7]. Nonetheless, agglomeration, arraying or networks of sensors combined with AI are expected to deliver a competitive alternative to spectrometers, sometimes compared to collective—or swarm—intelligence [8–10].

This article presents examples of sustainable processes from recycling of industrial process water, solvent, and plastics, from biotechnological production using disposable fed-batch reactors, and of product safety confirmation amenable to pharmaceutical production and release. The processes represent continuous, circular, and batch management. The applied PAT methodology comprises integral methods such as online solid-phase extraction ion-chromatography conductivity determination (SPE-IC-CD), impedance spectroscopy (IS) and specific methods such as mid-infrared (MIR), near-infrared (NIR) spectroscopy, and external cavity quantum cascade laser vibrational circular dichroism (EC-QCL-VCD) [5, 11–13]. On the basis of the reviewed examples, an outlook on future PAT developments will be ventured.

## PAT demands from closing process loops of waste water streams

**Fig. 1:**
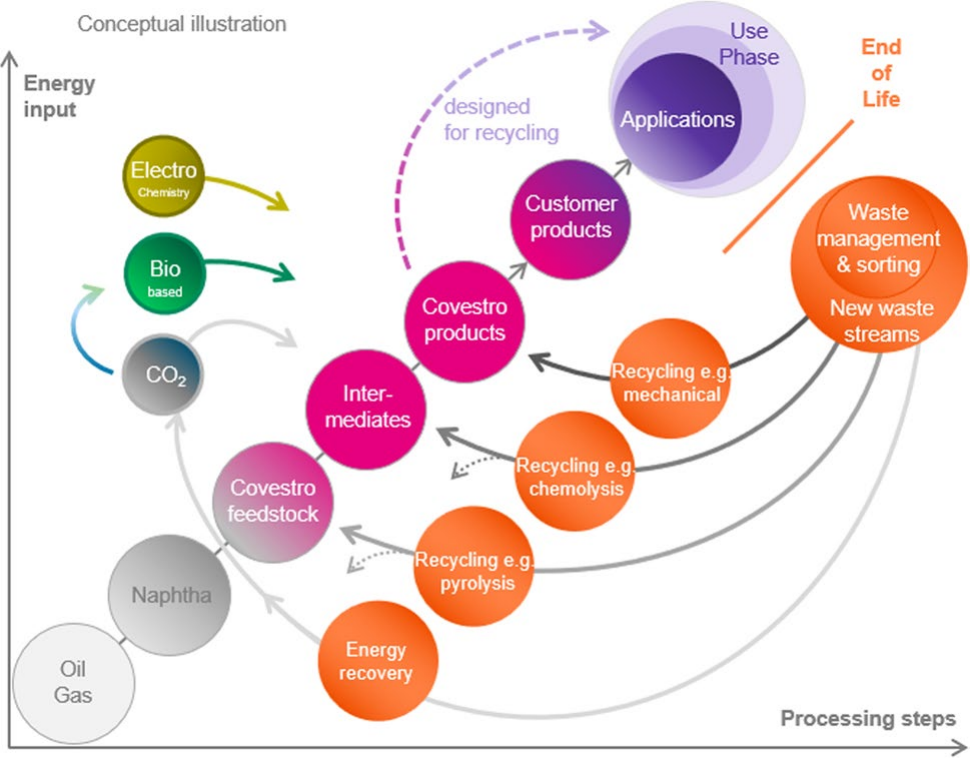
Toward a circular economy: Closing material and carbon loops as envisaged by Covestro; oil and gas-based processing steps (gray), biobased processes (green), and processes based on waste management and recycling (orange) [14]

A way toward sustainability and resource efficiency is realized to design the value chain of the—chemical—industry in a fully circular way. New processes with built-in circularity, see in Fig. 1, will need to shift away from oil and gas-based processing steps (gray) to biobased processes (green) and processes based on waste management plus recycling (orange).

For the process industry, becoming fully circular means building new processes that have circularity built-in and closing the material loops also in the existing brownfield plants. This is especially important within Europe and Germany, as the existing plant infrastructure should not be abolished for both economic and environmental reasons. Today, many of the large process industry hubs in Europe are operated as chemical Verbund, like in Ludwigshafen or Antwerp, where a by-product or waste stream from one plant is the substrate for the next plant. From a bird’s eye perspective, the process is still conducted in a rather linear way, albeit the Verbund integration. This includes carbon-based products and the indispensable by-streams such as salts or processed water. The resulting high salt concentrations in waste streams could be reused and would be a valuable resource. To fully utilize the water streams, the closed water cycle needs to fulfill the specifications of the corresponding downstream process. The chlorine-alkali (CA) electrolysis is one of the major industrial consumers of salt for the production of chlorine, hydrogen, and sodium hydroxide—essential building blocks for the manufacturing of numerous products. The highly saline aqueous streams could be recycled and fed into the CA electrolysis in case the high demands on the purity of the recycled aqueous stream are ensured, i.e., the salt concentration of the brine but also the concentration of specific organic molecules. The organic impurities of an upstream polymer production need to be controlled since they may lead to a voltage increase or damage of the electrolysis system. The resulting measurement task is to ensure detection in the ppb concentration and prevent specific components from reaching the CA electrolysis.

**Fig. 2:**
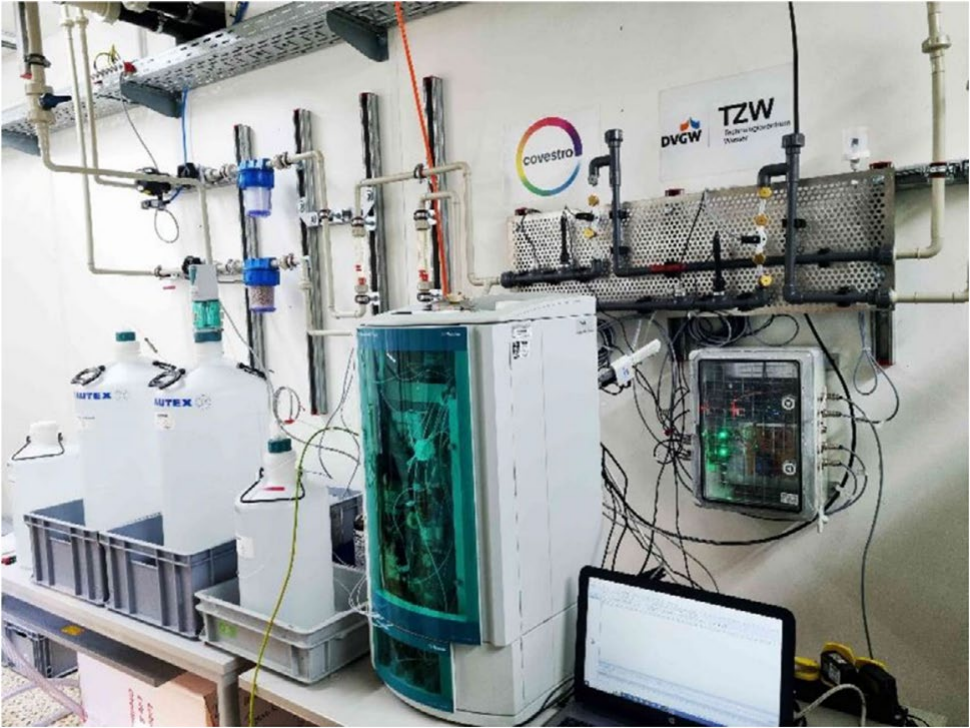
Custom-built online SPE-IC-CD system during the piloting at Covestro’s Uerdingen site

To solve the latter measurement task, a PAT concept was developed within the project RIKovery and piloted in a real-world application at the Covestro site in Uerdingen [15]. One of the critical process parameters was the concentration of a quaternary ammonium compound. The compound had to be removed upstream from the measurement position. To monitor the performance of the purification step, a method using ion chromatography in combination with conductivity detection was developed as an extractive online PAT system. Downstream from the first inline filtration step, solid phase extraction was added to the PAT system and sample preparation to reach the required low detection limits of less than 0.5 mg/L within the saltwater matrix containing 70–100 g/L of sodium chloride. The online SPE-IC-CD system was installed at several positions within the purification unit, cf. Figure 2, and was successfully tested over the course of 6 months. The combination of SPE and IC, one providing the matrix clean-up and analyte preconcentration and the other providing the separation, proved robust and easy to use. Its performance is necessary due to the complex matrices and low analyte concentrations. These conditions are often found in the recovery of valuable resources from otherwise waste streams but can be applied to the circular economy.

## Process NIR monitoring enables solvent recovery

**Fig. 3:**
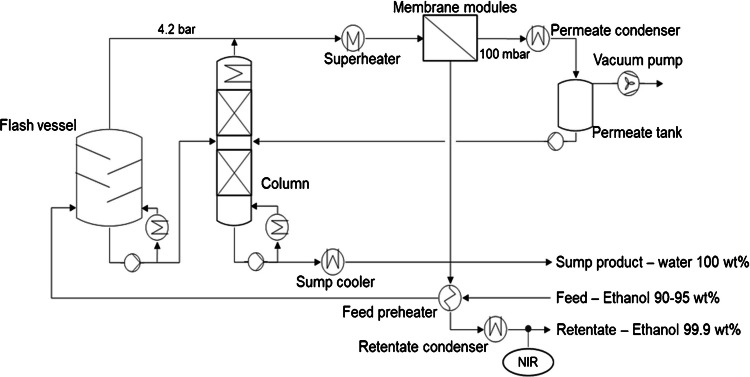
Simplified plant diagram for an ethanol recycling plant (courtesy of Bayer AG)

An illustrative example of the contribution of PAT to process solvent recycling is the ethanol recovery during the production of an active pharmaceutical ingredient (API) [16]. The reuse of ethanol contributes to circularity, economic efficiency, the CO_2_ footprint reduction, and environmental and climate protection [17]. The successful recovery yielded a rate of almost 100 percent. An ethanol–water mixture was received during API synthesis with the ethanol content typically varying between 90 and 95%. A large portion of the mixture was first evaporated in a flash tank and subsequently passed in gaseous form to a hydrophilic vapor permeation membrane of the membrane modules at a pressure of 4.2 bar, see Fig. 3. The sump of the flash was fed into a rectification column to recover the remaining ethanol. In the column, the remaining ethanol was recovered overhead as an azeotrope with a purity of approximately 95%. It was re-combined with the flash distillate toward the membrane modules.

When operated as intended, only water was contained at the bottom of the column. A few high boilers may be present as well, which were subsequently discharged and transferred to a wastewater treatment plant. Before reaching the hydrophilic permeation membrane, the distillates were superheated and conveyed as gas phase to the membrane modules. The resulting retentate consisted of denatured ethanol with a water content of less than 0.1 weight percent. To save energy, the feed was preheated by a partial condensation of the retentate. The retentate condenser liquefied the remaining ethanol, which was finally fed back as a solvent into the chemical reaction via a collecting tank.

**Fig. 4:**
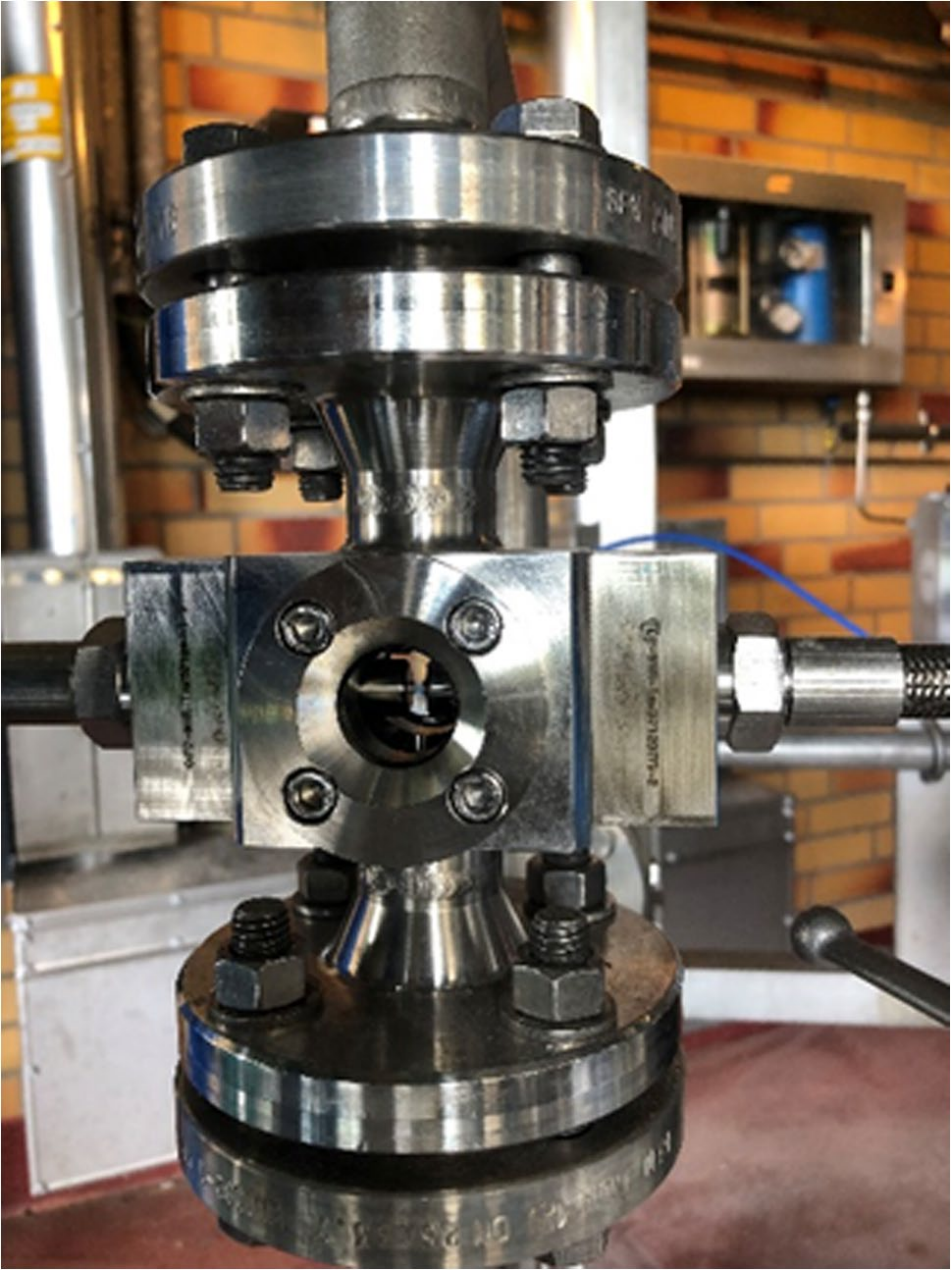
NIR probe implemented in a pipe of the ethanol recycling plant (courtesy of Bayer AG)

To monitor the ethanol quality, a NIR transmission flow probe was installed behind the retentate condenser, cf. Figure 3. An optical fiber to guide the NIR radiation connected the probe and a NIR-Fourier transform (FT) spectrometer equipped with an internal multiplexer and a halogen lamp, cf. Figure 4. The assembly was used for the continuous determination of the water content. To keep the maximum water content in the retentate below the specification of 0.1 weight percent, the recovery system feed was controlled based on the NIR analysis. The water content was calculated on the basis of a chemometric model using partial least squares (PLS) with a set of about 40 Karl-Fischer titration values as reference standards inside. The FT-NIR device provided a measurement value approximately every 40 s, which proved to be sufficient. To ensure fulfillment of the specifications, the maximum removable amount of water through the membrane modules determined the allowed water content. If the water content flowing to the membranes exceeded their separation capacity, the load was automatically reduced on the basis of the PAT monitoring data.

The contribution to circularity from emissions according to Scope 1, i.e., caused by Bayer directly, can be estimated at approximately 8800 t of CO_2_ per year due to the avoidance of ethanol solvent waste combustion [17]. As for Scope 3 emissions, which were not produced through Bayer AG, a saving of approximately 5750 t of CO_2_ equivalent per year has been reported. Furthermore, the economic benefits of ethanol recovery have grown in recent years due to the significant rise in the ethanol price.

In summary, a complete ethanol recovery of approximately 6000 t per year now leads to a significant CO_2_ footprint reduction. In particular, the use of NIR measurement technology for process control ensures cost-effectiveness by avoiding off-specification material, thus making a valuable contribution to our environment and climate neutrality.

## Developments in mid-infrared spectroscopy for material recognition as a sorting prerequisite

In industrial and municipal waste, different types and forms of plastics commonly occur. For their recycling and reuse, the type of plastic has to be identified and separated according to its chemical nature. The separation task demands a distinguishing feature of the different plastic species. It is of particular importance to recognize PVC, as it induces corrosion and hence poses a great danger for processing equipment. Non-plastic materials also need to be recognized in the waste, which is typically lumpy. Spectroscopic differentiation is a powerful means. Yet, the spectral range is to be chosen thoroughly, since many ranges are little suitable. Ultraviolet light-based spectroscopy, for example, is not sufficiently selective, and many plastics are difficult to identify due to additives. Differentiation is also hardly possible in the visible spectral range, since plastics are usually dyed and thus colorful for the needs of end consumers. The added organic dyes have defined chromophores in the visible range but do not absorb in the NIR and MIR range. Even for black-dyed textiles, the chromophores do no longer show absorption in the NIR range. Hence, NIR and MIR prove suitable ranges for analytical and monitoring tasks. So far, NIR spectroscopy has often been exploited, as fast detectors based on indium gallium arsenide pin diodes exist, many times realized as line spectrometers. Yet, the analytical monitoring systems need to be combined with multivariate data analysis, which requires extensive amounts of data and training at great expense. Due to the limited resolution and selectivity of NIR spectroscopy, different plastics display overlapping spectral bands, which cannot be analyzed in a univariate fashion. In spectroscopic terms, the MIR range is more favorable due to its extremely high selectivity both for plastics and other compounds and surfaces such as organic tissue, metals, and inorganic materials. Previous limitations were that surfaces could not be scanned simultaneously with sufficient selectivity, high contrast, and, above all, speed. Decisive advantages have been demonstrated in recent years [18].

**Fig. 5:**
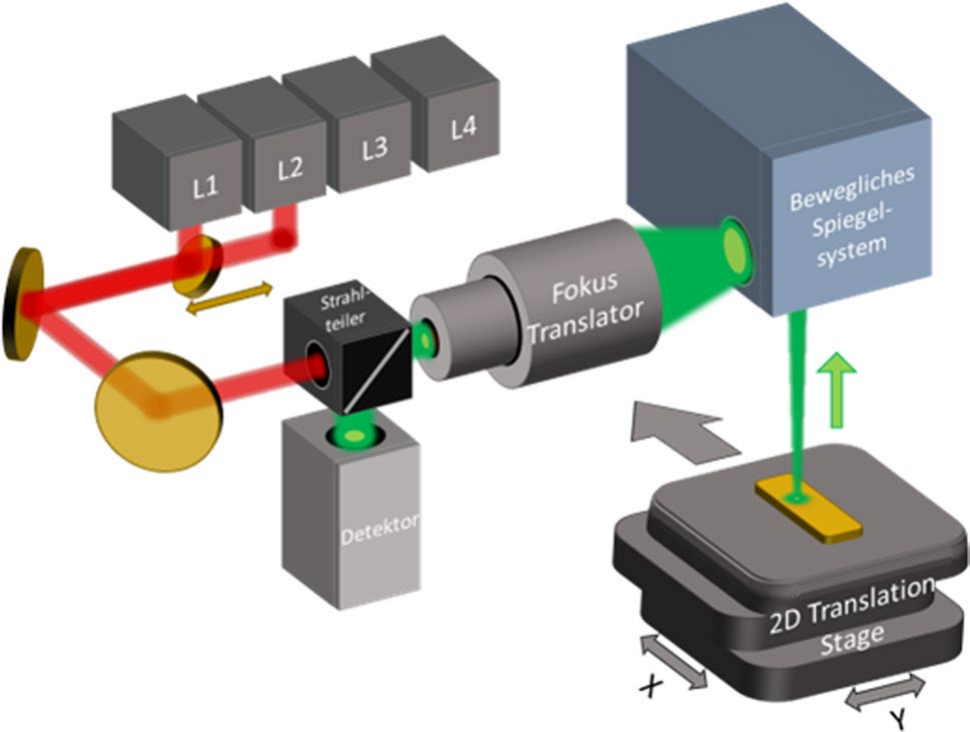
Fast-scanning IR unit with 3 × 106 points/sec for plastic detection [18]

Figure 5 shows a fast-scanning MIR assembly for plastic characterization in waste streams [18]. The device uses four wavelengths that are selected in such a way that the components of interest can be spectroscopically separated and hence identified simultaneously. The technology enables high-contrast surface detection. The scanning speed amounts to 3 × 10^6^ pixels per second with a spatial resolution of 10 µm. The performance allows detecting lumpy particles, plastic parts, and even coarse granules of around 50 µm in size. Once the components are characterized, mechanical separation is achieved by air flow. The air nozzles and the flow are controlled based on the results of the MIR monitoring.

In conclusion, the recovery of plastics as raw material will become more and more important in the future, since upcycling is a pillar of a circular economy. Fast-scanning MIR techniques will provide the high selectivity and surface identification for plastics and materials with sufficient speed to match the mechanical separation.

## PAT as a driver for sustainability in bioprocessing

Biotechnological processes play a crucial role in the production of pharmaceuticals, biofuels, and food ingredients. The shift from stainless steel to single-use technologies (SUT) has already enhanced sustainability by eliminating the need for cleaning-in-place (CIP) and sterilization-in-place (SIP), thereby reducing water and energy consumption. At the same time, it lowers the risk of cross-contamination and enables greater process flexibility, shorter batch turnaround times, a smaller footprint, and reduced capital investment [19].

Yet, single-use technology represents only one step toward more sustainable production. PAT further advances this trajectory by optimizing resource utilization and minimizing waste. Inline sensors in biopharmaceutical production are frequently used to monitor critical parameters such as pH, dissolved oxygen, and metabolites, ensuring efficient cell growth and reducing batch failures. Suitable metabolite sensors were realized as genetically encoded, electrochemical, optical, and enzymatic sensors. A real-time monitoring realization was reported to lead to up to 30% lower material consumption and reduced contamination risk [20, 21]. While pH and dissolved oxygen control are already highly automated and integrated into bioprocess workflows, the application of impedance spectroscopy and capacitance-based cell monitoring remains underexploited, with significant potential still to be unlocked [22–24].

Additionally, PAT was applied to enhance process efficiency in fed-batch and perfusion systems, where precise control of nutrient feed rates minimized excess media usage while maintaining optimal cell viability. This not only lowered raw material consumption and extended culture longevity but also reduced the overall environmental footprint. In perfusion processes, process times were shortened, material use was reduced, and costs were lowered. At the same time, higher cell densities were achieved, resulting in increased product yield and quality—all within a smaller production volume [25]. PAT thus offers a complementary approach that strengthens both efficiency and sustainability. Other industries also leverage PAT for sustainability. In biofuel production, PAT-driven fermentation control increased ethanol yields while minimizing feedstock waste [26]. The food industry has benefited from near-infrared (NIR) spectroscopy to optimize ingredient use, reducing food waste and energy consumption [27].

Looking ahead, the role of PAT is expected to expand even further. Emerging technologies such as artificial intelligence (AI), machine learning, and digital twins are increasingly being integrated with PAT systems, allowing for predictive control strategies and real-time decision making. These developments will open new possibilities for adaptive bioprocessing, in which systems will autonomously respond to changes in process conditions to maintain optimal performance [28]. Moreover, the continued miniaturization and modularization of sensor technology will support decentralized and agile manufacturing concepts, aligning with the growing demand for flexible, small-scale production units in personalized medicine and on-demand biologics manufacturing [21].

## External cavity quantum cascade laser vibrational circular dichroism for enantiomeric discrimination

Due to the distinct pharmacological effects of enantiomers, the characterization of chiral pharmaceuticals has been mandated by the Food and Drug Administration (FDA) since 1992 and the European Medicines Agency (EMA) since 1994 [29]. This has driven advancements in asymmetric synthesis and chiral analytical techniques to monitor and ensure enantiomeric purity in both production and quality control. However, conventional chiral analytical methods such as X-ray crystallography and nuclear magnetic resonance (NMR) are time-intensive and unsuitable for real-time process monitoring, making them largely incompatible with PAT requirements.

**Fig. 6:**
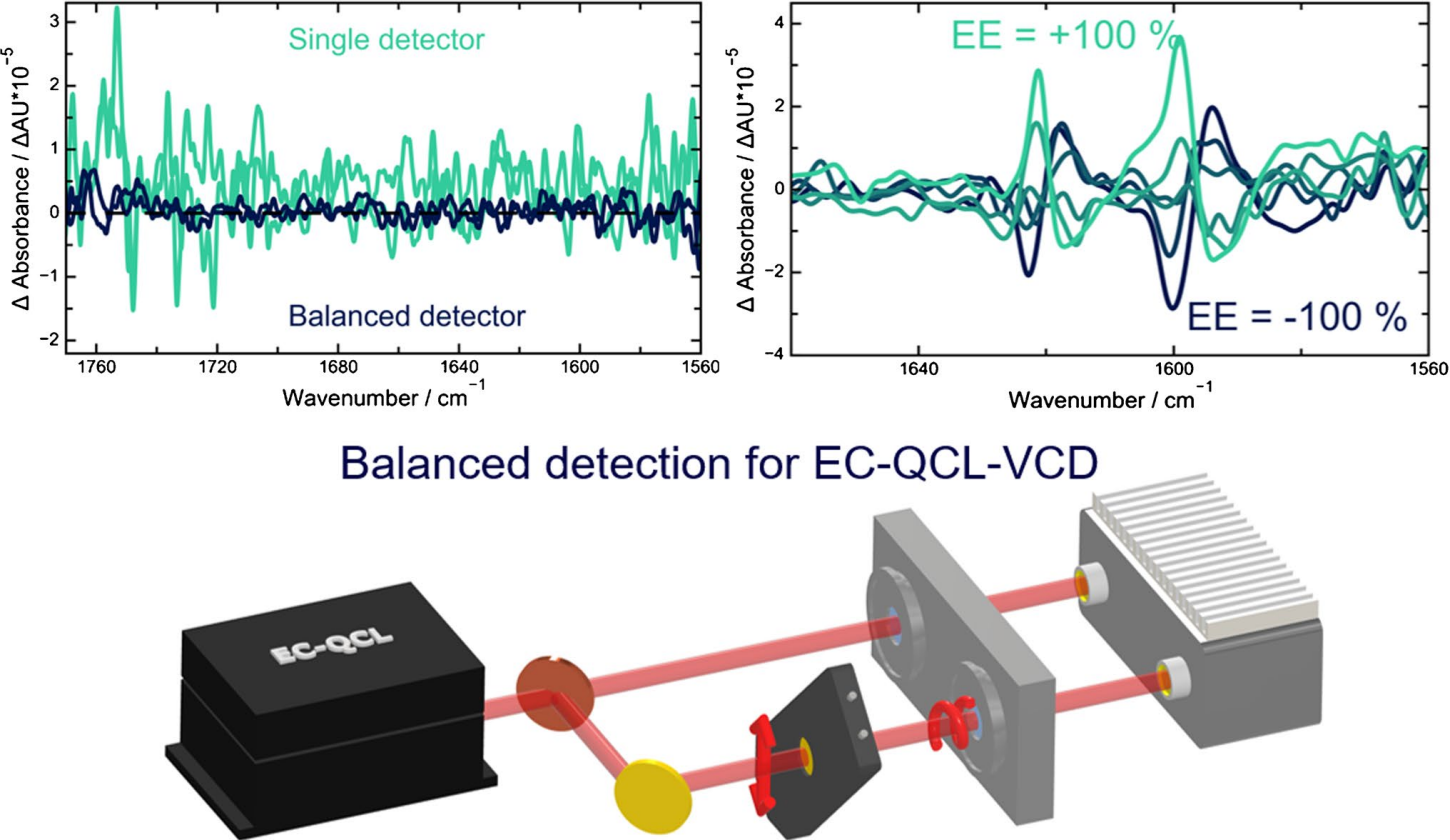
Simplified scheme of the instrument for the balanced VCD measurements using an EC-QCL as a light source (bottom). Conventional and balanced VCD spectral noise levels (top left) and VCD spectra of a (S)-(-)- and (R)-(+)−1,1′-bi-2-naphthol (top right) from ref. [30] CC-BY 4.0

Vibrational circular dichroism is a powerful technique for determining the absolute configuration of molecules without the need for specialized reagents or chiral separation columns, based on differential absorption of left- and right-circularly polarized light in the infrared spectral region. However, VCD signals are typically 4–6 orders of magnitude weaker than classical absorbance signals, requiring long acquisition times to achieve acceptable signal-to-noise ratios (SNRs). By utilizing the high brilliance of an external cavity quantum cascade laser (EC-QCL) combined with enhanced stabilization via a two-detector balanced detection scheme, cf. Figure 6. Similar noise levels are achieved as compared to state-of-the-art FT-IR-VCD in significantly shorter acquisition times [30].

This advancement enabled initial studies on small molecules and proteins [13, 31]. Recently, EC-QCL-VCD with an acquisition time of 146 s was demonstrated for real-time monitoring of enantiomeric excess (EE) of the enantiomeric pair R/S-1,1′-bi-2-naphthol in solution. Different mixtures of the two components were used to simulate a racemization process. Spectral data were collected at a time resolution of 6 min over three concentration levels. QCL-VCD was evaluated in terms of both molar and enantiomeric excess (EE) sensitivity at a time resolution relevant to chiral monitoring in chemical processes. Subsequent chemometric evaluation by partial least squares regression revealed a cross-validated prediction accuracy of 2.8% EE with a robust prediction also for the test data set (error = 3.5% EE). A key advantage of EC-QCLs is their broad tunability, while simultaneously enabling the precise selection of individual wavenumbers for measurements. This characteristic not only provides exceptional flexibility but also enables significantly faster VCD measurements compared to conventional FT-IR-VCD. This capability has proven effective in secondary structure elucidation of proteins, enabling detection at concentrations as low as 10 mg/mL in D_2_O within 5 min by restricting emission to the amide I′ band. Such enhanced time resolution facilitates real-time monitoring of protein dynamics via VCD [13].

A major challenge in achieving highly sensitive, high-time-resolution EC-QCL-VCD is the minimization of linear birefringence, which introduces baseline artifacts due to polarization state alterations. These artifacts primarily arise from optical imperfections, such as strain in lenses and windows. To mitigate these effects, highly optimized optical systems and advanced multi-modulation techniques, such as dual polarization modulation, are required [32].

While VCD provides valuable insights into a wide range of analytes, its use has been limited by the challenges of FT-IR-VCD. Modern QCL technologies such as broadly tunable EC-QCLs or arrays of discrete wavelength DFB-lasers offer a transformative solution to overcome these limitations. The higher throughput and the possibility to concentrate on the most informative wavelengths only, along with innovative spectroscopic designs, will allow for a further increase in sensitivity, leading to significantly reduced measurement times for targeted applications. Based on the QCL technology, the measurement time to obtain high-quality VCD spectra is thus reduced from hours to a few minutes. This will most likely extend VCD’s applicability beyond fundamental research, paving the way also for online chiral reaction monitoring. It has the potential to excel in real-time determination of EE, reducing the importance of time-consuming off-line chiral chromatography for process monitoring and control. This will reduce waste and cycle times and thus contribute to sustainable processes. Furthermore, we see great potential in the use of EC-QCL-VCD for the study of protein structural changes and protein activity in biotechnological processes.

## Expert opinion

The new process technologies needed to close the envisaged circular material streams will demand new PAT solutions for new measuring tasks. Some will mean new pathways to companies, and some will define new measuring tasks using existing PAT—but others will lead to unsolved PAT tasks to be solved. Among the latter, the first step of plastics recycling, i.e., material recognition, is a sorting prerequisite. Techniques such as vibrational spectroscopy provide the desired high specificity and speed. The future of PAT also faces significant challenges in enabling circularity in water and wastewater applications where trace component detection is crucial. These measurement tasks will require detection capabilities in the ppb range and at molecular level specificity. Since most online methods lack either the required sensitivity or specificity, online chromatographic methods and online sample preconcentration techniques such as SPE-IC-DC will become increasingly important to address these PAT challenges. Additionally, regulatory requirements will continue to shape the development of future PAT tasks, emphasizing the need for highly accurate and reliable measurement technologies. For pharmaceutical processes, sophisticated methods such as EC-QCL-VCD to distinguish enantiomers are promising advancements. The high composition variability of biobased feedstocks and substrate streams from recycling has a severe impact on the downstream processing or may render certain downstream processes hardly feasible at an industrial level unless PAT monitors and assists to reduce this variability. In this context, PAT does not merely serve as a tool for optimization—it becomes a strategic pillar in building resilient, future-ready manufacturing systems that are both economically viable and environmentally responsible. As sensitivity and especially throughput of the novel sensing techniques continue improving, machine learning (ML) algorithms will be applied more and more to convert raw spectra and data to chemical information. Modern deep learning approaches, e.g., convolutional neural networks, offer a promising way forward expanding on the capabilities of multivariate calibrations. Yet, it has to be kept in mind that a model is only as good as the data used for training. If the artificially augmented training data ignore or contradict basic physical laws, this will also apply to the model based thereon. Thus chemical and spectroscopic expertise—today often referred to as domain expertise—will remain fundamental for accurate quantitative analysis, even while large language model (LLM) driven automation increasingly hides the underlying complexity and facilitates the establishment of chemometric models for non-expert users. This is supported by the facile combination of readily available LLM and Python. Skilled applications have been demonstrated in the petrochemical and the dairy industry, where the feedstock of raw materials varies, and the automated generation of models is a great progress [33–35]. As industries continue to advance digitalization and automation, PAT remains a key enabler of sustainable, high-quality bioprocessing. Sensors have established themselves as low-cost, fast-acquisition tools, while their combination to swarms has yet to be exploited. It remains of prime importance that knowledge transfer is encouraged through sectors and branches. By integrating PAT across industries, manufacturers have not only improved process efficiency but also contributed to the UN Sustainable Development Goals (SDGs), particularly Responsible Consumption and Production (SDG 12) and Climate Action (SDG 13). One of the key topics for success remains to accompany the new processes from the lab phase via piloting to large scale production units with PAT [36]. Its widespread adoption will be essential in achieving the dual goals of industrial competitiveness and planetary health.

## Data Availability

No new data were created or analyzed in this study. Data sharing is not applicable to this article.
